# Supplementary material for: Working during a Pandemic between the Risk of Being Infected and/or the Risks Related to Social Distancing: First Validation of the SAPH@W Questionnaire
Source: Int J Environ Res Public Health. 2021 Jun 2;18(11):5986. doi: 10.3390/ijerph18115986 (PMC8199693; doi:10.3390/ijerph18115986)
Supplement: Supplementary file 1 [file ijerph-18-05986-s001.zip › ijerph-1224383-supplementary.pdf]

International Journal of
Environmental Research and Public Health

Table S1. Latent mean differences: Gender, ecological risk level, and type of occupation.

	**Gender: Female** [a]		**Ecological risk level: High-risk zone** [b]		**Type of occupation: Remote working** [c]	
	**Mean**	***SE***	**Mean**	***SE***	**Mean**	***SE***
Communication	-0.118	0.082	-0.012	0.082	-0.209 **	0.080
Decision-making	-0.165	0.089	-0.164	0.089	-0.177 *	0.089
Situational awareness	-0.173 *	0.087	-0.019	0.087	-0.190 *	0.087
Fatigue management	-0.174	0.093	-0.245 **	0.093	-0.157	0.095
Personal contribution	-0.191 **	0.068	-0.071	0.068	-0.096	0.068

Note: SE is standard error; [a]−The reference group for gender was male; [b]−The reference group for ecological risk level was low-risk zone; [c]−The reference group for type of occupation was in person; **p* < .05. ***p* < .01.
